# Association of *MTHFR* gene polymorphisms with non-Hodgkin lymphoma risk: Evidence from 31 articles

**DOI:** 10.7150/jca.99351

**Published:** 2024-08-13

**Authors:** Gang Wang, Yuluo Wu, Zuolei Jing, Ruiting Wen, Yuanrui Song, Yin Feng, Guangru Li, Xiaopeng Zou, Gaoxiang Huang, Zhirong Jia, Yunmiao Guo, Zhigang Yang

**Affiliations:** 1 Clinical Research Institute of Zhanjiang, Central People's Hospital of Zhanjiang, Guangdong Medical University Zhanjiang Central Hospital, Zhanjiang 524045, P. R. China.; 2 Department of Oncology, Central People's Hospital of Zhanjiang, Guangdong Medical University Zhanjiang Central Hospital, Zhanjiang 524045, P. R. China.; 3 Department of Hematology, Central People's Hospital of Zhanjiang, Guangdong Medical University Zhanjiang Central Hospital, Zhanjiang 524045, P. R. China.; 4 Zhanjiang Key Laboratory of Leukemia Pathogenesis and Targeted Therapy Research, Central People's Hospital of Zhanjiang, Guangdong Medical University Zhanjiang Central Hospital, Zhanjiang 524045, P. R. China.

**Keywords:** *MTHFR*, C677T, A1298C, polymorphism, susceptibility

## Abstract

**Background:*** Methylenetetrahydrofolate reductase* (*MTHFR*) gene polymorphisms, particularly C677T and A1298C, have been implicated in various cancers, including non-Hodgkin lymphoma (NHL); however, their association with NHL risk remains inconclusive.

**Methods:** We conducted an updated meta-analysis to assess the relationship between *MTHFR* gene polymorphisms (C677T and A1298C) and NHL risk. Relevant studies were identified through systematic literature searches in multiple databases. Pooled odds ratios (ORs) with 95% confidence intervals (CIs) were calculated to evaluate the strength of the associations.

**Results:** The meta-analysis included 32 studies (8222 cases vs. 12956 controls) for *MTHFR* C677T and 26 studies (6930 cases vs. 11611 controls) for the A1298C polymorphism. Our meta-analysis revealed no significant associations between *MTHFR* gene polymorphisms (C677T and A1298C) and NHL risk. However, subgroup analysis stratified by ethnicity and NHL subtype yielded interesting findings for the C677T polymorphism. Specifically, in the subgroup analysis of Caucasians, the C677T polymorphism was significantly associated with NHL risk (heterozygous: OR=1.16, 95% CI=1.02-1.32; allele comparison: OR=1.07, 95% CI=1.01-1.13). Furthermore, in the analysis stratified by NHL subtype, the C677T polymorphism was significantly associated with increased follicular lymphoma (FL) risk (homozygous: OR=1.25, 95% CI=1.02-1.53; recessive: OR=1.28, 95% CI=1.06-1.56). False-positive result possibility (FPRP) analysis verified that the association of the *MTHFR* C677T polymorphism with NHL risk for Caucasians and FL subtypes was a true positive and deserves attention. We also determined that the C677T polymorphism is an expression quantitative trait locus (eQTL) since it is associated with *MTHFR* gene expression.

**Conclusion:** There was no overall association between *MTHFR* gene polymorphisms (C677T and A1298C) and NHL risk, but stratified analyses revealed significant associations in specific subgroups. While meta-analyses inherently build upon existing studies, our work distinguishes itself by incorporating recent data, applying rigorous analytical techniques, and providing more evidence of the *MTHFR* C677T polymorphism as an eQTL.

## Introduction

Non-Hodgkin lymphoma (NHL), which originates in the lymphatic system, is a complex group of blood cancers with more than 50 different subtypes and is classified mainly into B-cell, T-cell, and natural killer (NK)-cell lymphomas on the basis of the type of lymphocyte affected 1. Some of the most common types of NHL include diffuse large B-cell lymphoma (DLBCL), follicular lymphoma (FL), mantle cell lymphoma, and peripheral T-cell lymphoma 2. The severity of high-grade NHL may require patients to undergo combination therapy, including chemotherapy, immunotherapy, and radiotherapy. In contrast, indolent lymphomas are usually incurable but are best managed as a lifelong, chronic disease. According to global cancer statistics from 2022, NHL ranks 10^th^ in incidence and 11^th^ in mortality 3. Many lifestyle factors, environmental factors, and genetic factors, including smoking, alcohol consumption, hair dye, ultraviolet radiation, occupational exposure, immune deficiency, and micronutrients involved in one-carbon metabolism (e.g., B6, B12, methionine, and folate), have been shown to be associated with NHL risk; however, the risk may vary among NHL subtypes 2, 4-7. Mounting evidence indicates that adequate intake of folate protects alcohol abstainers and former alcohol drinkers from developing NHL 4 and reduces the risk of DLBCL and marginal zone lymphoma 8.

Folate metabolism is closely intertwined with one-carbon metabolism. Folate, a B vitamin, is a crucial component of one-carbon metabolism. In folate metabolism, 5,10-methylenetetrahydrofolate reductase, encoded by the *MTHFR* gene, converts 5,10-methylenetetrahydrofolate into biologically active 5-methyltetrahydrofolate. The resulting active form of folate transfers carbon units to acceptor molecules through a series of enzymatic reactions (e.g., the conversion of homocysteine to methionine), completing the one-carbon transfer process. During one-carbon metabolism, the carbon units provided by 5-methyltetrahydrofolate are transferred and utilized to synthesize various biomolecules, including nucleic acids, amino acids, lipids, and neurotransmitters. Additionally, one-carbon metabolism is involved in methylation reactions (e.g., DNA CpG island methylation), which play critical roles in cellular differentiation, gene expression regulation, and other biological processes 9, 10. Therefore, defects in the *MTHFR* gene disrupt multiple fundamental biochemical processes, including cell cycle regulation, DNA replication, and DNA and protein methylations, leading to various disorders, such as neural tube defects, cancer, and cardiovascular diseases 11-13. Accordingly, genomic DNA methylation positively correlates with plasma folate 10. Research has shown that two polymorphisms in the *MTHFR* gene, C677T and A1298C, can reduce enzyme activity 9, 10. The *MTHFR* C677T variant leads to decreased intracellular methylation reactions, with the T/T genotype of *MTHFR* C677T dictating genomic DNA hypomethylation, a feature of most cancers 10, 14.

Polymorphisms in this gene have been studied with respect to the risk of various cancers, including NHL. Over the past few decades, many studies have investigated the associations between two *MTHFR* polymorphisms (C677T and A1298C) and NHL risk. However, the inconsistencies or limitations in the literature are not ignorable. Initial meta-analyses provided some insights but were limited by small sample sizes, regional biases, and variations in study quality 15. By including a broader array of recent studies, the aim of this study was to conduct a systematic and updated meta-analysis to reassess the association between *MTHFR* polymorphisms and NHL risk to help elucidate the genetic underpinnings of NHL and offer potential preventative strategies.

## Materials and methods

### Sources

Literature searches were conducted via PubMed and EMBASE to collect studies on the association of *MTHFR* polymorphisms with NHL. The search employed the keywords “*MTHFR* or methylenetetrahydrofolate reductase”, ''polymorphism or variant or variation'', and ''non-Hodgkin lymphoma or non-Hodgkin's lymphoma or NHL”, coupled with the term “dependence” (the last search updated was on April 18, 2024). The Chinese Biomedical Literature Database (CBM) was also screened via the same search strategy to identify publications written in Chinese. We subsequently reviewed the bibliographies of the articles captured through the electronic search to find additional articles. We analysed studies that measured the association between *MTHFR* polymorphisms and NHL. Specifically, we were interested in studies of *MTHFR* polymorphisms known to affect the enzyme activity of *MTHFR*, C677T (Ala222Val, rs1801133) and A1298C (Glu429Ala, rs1801131).

### Inclusion and exclusion criteria of studies

Studies eligible for the final meta-analysis needed to (1) investigate *MTHFR* C677T and/or A1298C polymorphisms in relation to NHL risk; (2) be structured as case‒control, nested case‒control, or cohort studies; (3) be published in English or Chinese; (4) be available for single nucleotide polymorphism (SNP) genotype data; (5) be distinct from other studies, with no overlapping datasets; and (6) supply adequate data to determine ORs and 95% CIs. The exclusion criteria applied to studies were the control genotype frequencies for the *MTHFR* C677T and A1298C polymorphisms did not adhere to Hardy‒Weinberg equilibrium (HWE) or lacked additional verification of HWE for other SNPs. Additional exclusions included case-only studies, case reports, conference abstracts, reviews, meta-analyses, and studies without adequate data. If there were two or more case‒control studies involving the same subjects, we included only the newest study or the study with the largest sample size in the final meta-analysis.

### False-positive report probability analysis (FPRP)

FPRP is a statistical method that helps determine the probability that a statistically significant result is a false-positive, considering certain assumptions about prior probabilities of a true association for each finding and the observed data. We employed FPRP analysis to assess the robustness of statistically significant findings for the current genetic association studies. We calculate the FPRP for each significant finding via the observed *P* value, the prior probability (0.25, 0.1, 0.01, 0.001, or 0.0001), and the study's statistical power.

### Expression quantitative trait locus analysis

An expression quantitative trait locus (QTL) is defined as a genetic variant that is significantly correlated with nearby gene expression alterations. The Adult Genotype Tissue Expression (GTEx) project, launched in 2010, is a large-scale research effort to understand the genetic regulation of gene expression in human tissues. GTEx collects and analyses genetic data and tissue samples from deceased adult donors across diverse populations in the United States. The project has generated extensive datasets and resources that are freely accessible to the scientific community, facilitating the study of how genetic variations influence gene expression patterns in various tissues 16. We used this GTEx web tool (www.gtexportal.org/) to explore whether the *MTHFR* SNPs affect gene expression.

### Statistical analysis

We calculated ORs and 95% CIs to estimate the associations between *MTHFR* SNPs and NHL susceptibility. We evaluated the risk of developing NHL for the assumed underlying genetic models, including the homozygous model (C677T: TT vs. CC; A1298C: CC vs. AA), heterozygous model (C677T: CT vs. CC; A1298C: AC vs. AA), recessive model (C677T: TT vs. CT+CC; A1298C: CC vs. AC+AA), and dominant model (C677T: CT +TT vs. CC; A1298C: AC+CC vs. AA). Allele comparisons were also performed to appraise the risk of mutant alleles over wild-type alleles for the two SNPs (C677T: T vs. C; A1298C: C vs. A). The goodness-of-fit chi-square test was applied to assess the departure from Hardy-Weinberg equilibrium (HWE) in the control genotypes. Significance was determined at a level of *P*<0.05. We examined the heterogeneity among the studies via the chi-square-based Q test. In cases where significant heterogeneity was present (*P*_heterogeneity_<0.10), a random-effects model was selected 17, whereas a fixed-effects model (the Mantel-Haenszel method) was applied otherwise 18. Identifying sources of heterogeneity is crucial for interpreting the overall results of a meta-analysis and can guide future research by highlighting areas where further investigation is warranted. To address heterogeneity, we examined whether the association varies across different subgroups stratified on the basis of ethnicity (Asians, Caucasians, Africans, and Mixed groups), source of control (hospital-based and population-based), and tumor subtype (FL and DLBCL). A sensitivity analysis was conducted to assess the stability of the findings, systematically excluding one study at a time and reiteratively computing the pooled ORs and 95% CIs. To investigate potential publication bias, both Begg's funnel plot 19 and Egger's linear regression test 20 were executed. All the statistical analyses were carried out via STATA software (version 11.0; Stata Corporation, College Station, TX) and SAS software (version 9.1; SAS Institute, Cary, NC). Significance was assessed via two-sided tests, with *P*<0.05 indicating statistical significance.

## Results

### Study characteristics

**Figure 1:**
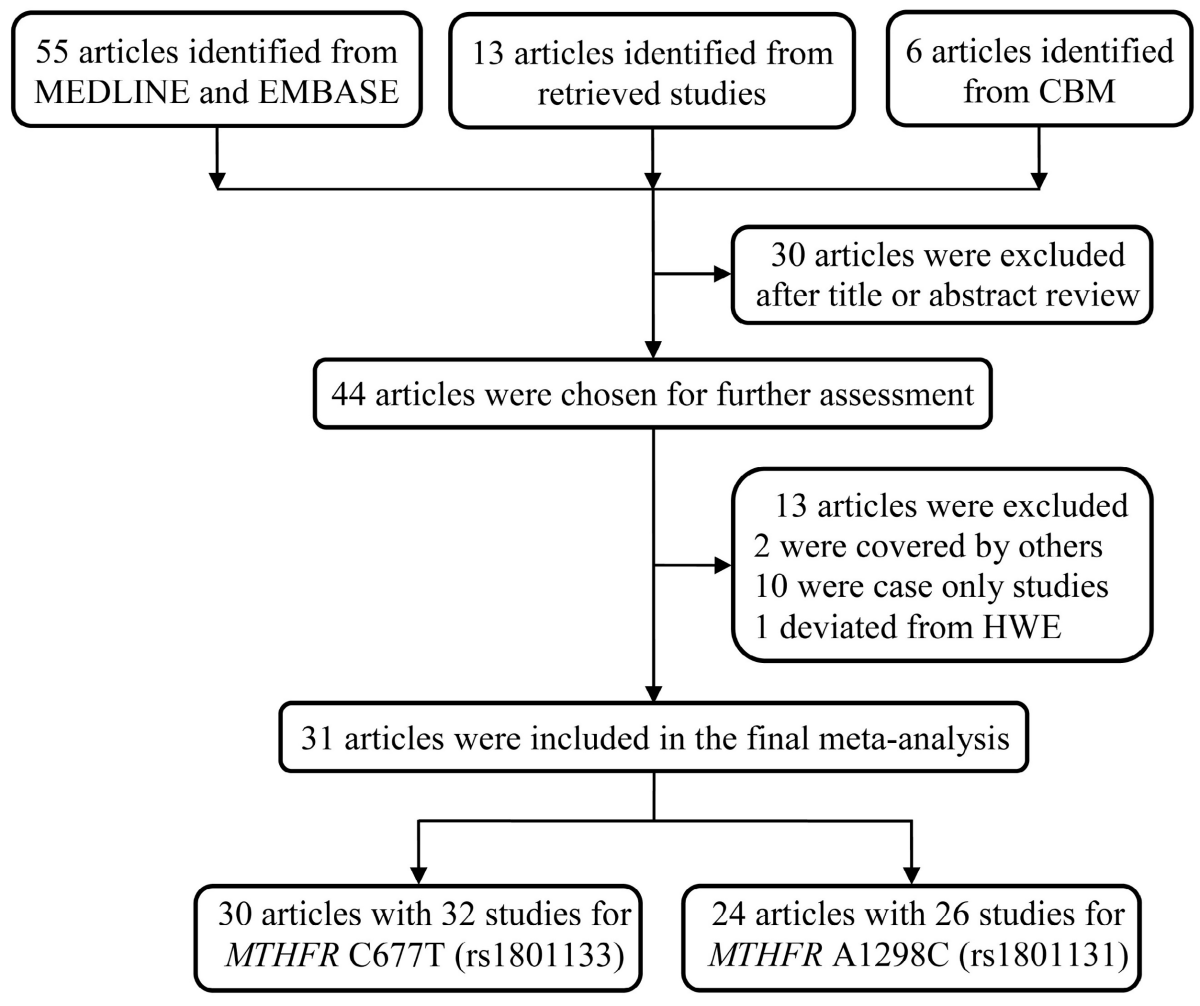
The schematic diagram of the article screening process for the meta-analysis.

We initially identified 74 studies regarding the association between *MTHFR* polymorphisms and NHL susceptibility. After reviewing the title and abstract, we excluded 30 articles, including reviews and studies not involving the SNPs C677T and A1298C. An additional 13 articles were discarded since they overlapped with the included studies, were case-only studies, or deviated from HWE. As a result, 31 articles were selected for the final meta-analysis, with all the samples in the studies in HWE (**Table 1**) 9, 12, 13, 21-46. All these studies adopted a case‒control design. Among them, 30 articles consisted of 32 studies that compared the frequency of *MTHFR* C677T alleles in NHL patients and controls, whereas 24 articles with 26 studies focused on the association between the *MTHFR* A1298C polymorphism and NHL risk (**Figure 1**).

### Meta-analysis results

The association between the *MTHFR* polymorphism and the risk of NHL was recapitulated in 31 articles, consisting of 32 case‒control studies for the SNP rs1801133 (C677T) and 26 studies for the SNP rs1801131 (A1298C). The characteristics of the relevant case‒control studies evaluating the SNPs rs1801133 and rs1801131 (A1298C) are shown separately in **Table 1**.

**Figure 2:**
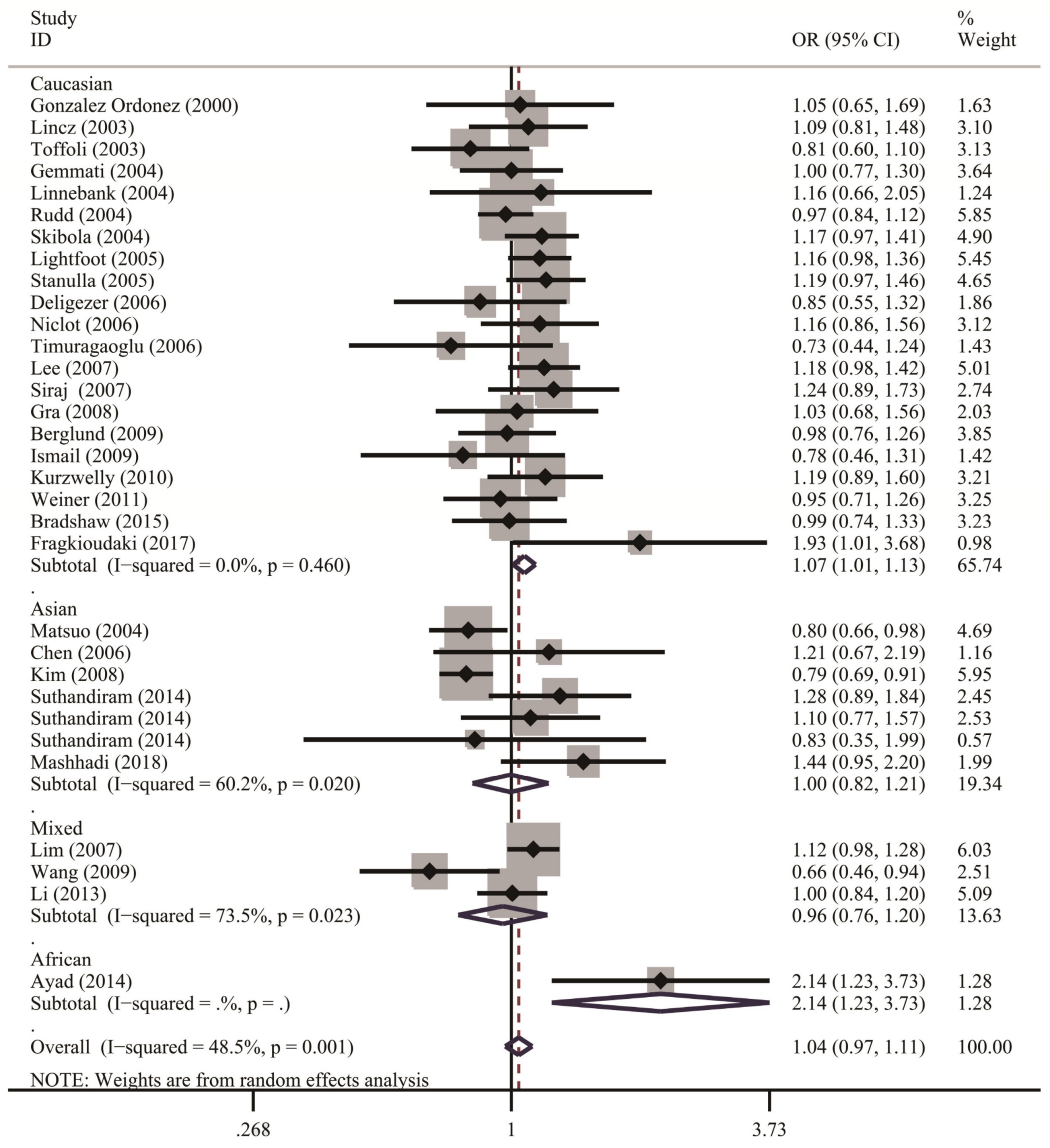
Forest plots demonstrate the association between *MTHFR* C677T polymorphisms and NHL risk in the stratified analysis by ethnicity regarding allele comparison. A solid diamond shape and a horizontal line on the plot visually represent the estimation of OR and its 95% CI for each study.

**Figure 3:**
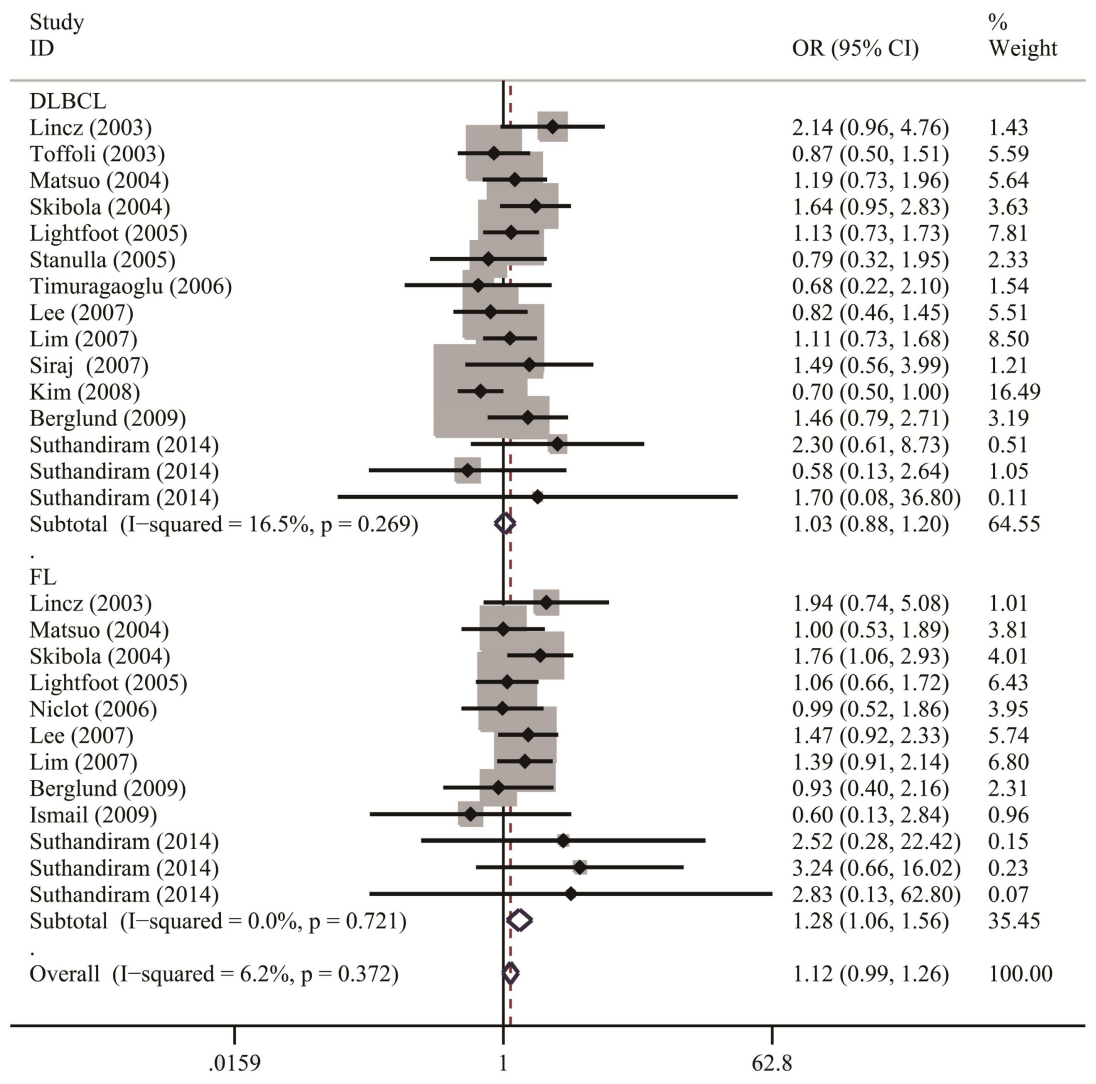
The forest plots illustrate the relationship between *MTHFR* C677T polymorphisms and the risk of NHL under the recessive model, segmented by NHL subtypes. Each study's OR and its 95% CI are graphically presented using a solid diamond shape and a horizontal line for visual clarity.

**Table 2** lists the results of pooled and stratified analyses for the two SNPs. The pooled ORs and 95% CIs revealed that no association existed between the *MTHFR* C677T polymorphism and susceptibility to NHL across the 32 studies included in the analysis (homozygous: OR=1.10, 95% CI=0.96-1.24; heterozygous: OR=1.00, 95% CI=0.92-1.10; recessive: OR=1.06, 95% CI=0.97-1.17; dominant: OR=1.02, 95% CI=0.94-1.12; allele comparison: OR=1.04, 95% CI=0.97-1.11). Given that studies in the meta-analysis vary regarding population characteristics, tumor subtypes, and methodologies, stratified analysis may provide more informative guidance than overall analysis does and allow us to examine how these differences might affect the overall results. In the analysis stratified by ethnicity (**Figure 2**), the pooled OR under the homozygous model for the Caucasian subgroup was 1.16 (95% CI=1.02-1.32), with a Q statistic indicating heterogeneity (*P*=0.626). Allele comparison further provided evidence that the T variant allele is a risk factor for NHL in Caucasians (OR=1.07, 95% CI=1.01-1.13). Moreover, the T variant allele appeared to greatly increase the NHL risk in Africans (heterozygous: OR=2.91, 95% CI=1.34-6.32; dominant: OR=2.89, 95% CI=1.39-6.00; allele comparison: OR=2.14, 95% CI=1.23-3.73). However, this ethnic group included only one study with 49 cases and 82 controls. The source of control did not affect the significance of the association with NHL risk. In addition, stratified analysis by NHL subtype (**Figure 3**) revealed that carriers of the *MTHFR* C677T TT genotype were at significantly greater risk of developing FL (homozygous: OR=1.25, 95% CI=1.02-1.53; recessive: OR=1.28, 95% CI=1.06-1.56) than those with the CT and/or CC genotypes were (**Table 2**).

Like the *MTHFR* C677T polymorphism, in the 26-study pooled analysis, we found no significant association between the *MTHFR* A1298C polymorphism and overall NHL risk (homozygous: OR=1.20, 95% CI=0.99-1.47; heterozygous: OR=1.00, 95% CI=0.94-1.07; recessive: OR=1.20, 95% CI=1.00-1.44; dominant: OR=1.04, 95% CI=0.95-1.13; allele comparison: OR=1.07, 95% CI=0.98-1.17). The same applied to the stratified analysis for the A1298C polymorphism by ethnicity, source of control, and NHL subtypes (**Table 2**).

### Heterogeneity and sensitivity analyses

The Q test revealed the presence of substantial heterogeneity in the association between the two *MTHFR* SNPs and NHL susceptibility, particularly in the overall analysis (**Table 2**). This finding suggested variability in the meta-analysis outcomes beyond what would be expected owing to chance alone. However, subgroup analyses indicated that heterogeneity was attenuated in Caucasians and in the FL subgroup (**Table 2**). Sensitivity analyses conducted by iteratively removing one study at a time revealed that none of the individual studies had a notable effect on the overall ORs (data not shown).

### Publication bias

We checked the potential bias of the meta-analysis via Begg's funnel plot 19 and Egger's linear regression test 20. Asymmetry was observed in the shape of the funnel plots concerning the C677T and A1298C polymorphisms (data not presented). Moreover, Egger's test did not indicate any significant publication bias for either the C677T or A1298C polymorphisms. These results suggested that this meta-analysis was not influenced by publication bias.

### FPRP results

While assuming a prior probability of 0.25, low FPRP values were yielded for the significant association between the *MTHFR* C677T polymorphism and NHL risk among the following subgroups: Caucasian (TT vs. CC, 0.062; T vs. C, 0.065), FL subtype groups (TT vs. CC, 0.092; TT vs. CT/CC, 0.033), and African (T vs. C, 0.141) (**Table 3**). These findings with low FPRP values indicate a high probability that the associations are true positives and are robust against false-positives. When a stricter prior probability of 0.1 was applied, the associations for the Caucasian (TT vs. CC, 0.165; T vs. C, 0.171) and FL subtype groups (TT vs. CT/CC, 0.094) remained noteworthy (**Table 3**).

### Genotype‒tissue expression (GTEx) analysis

**Figure 4:**
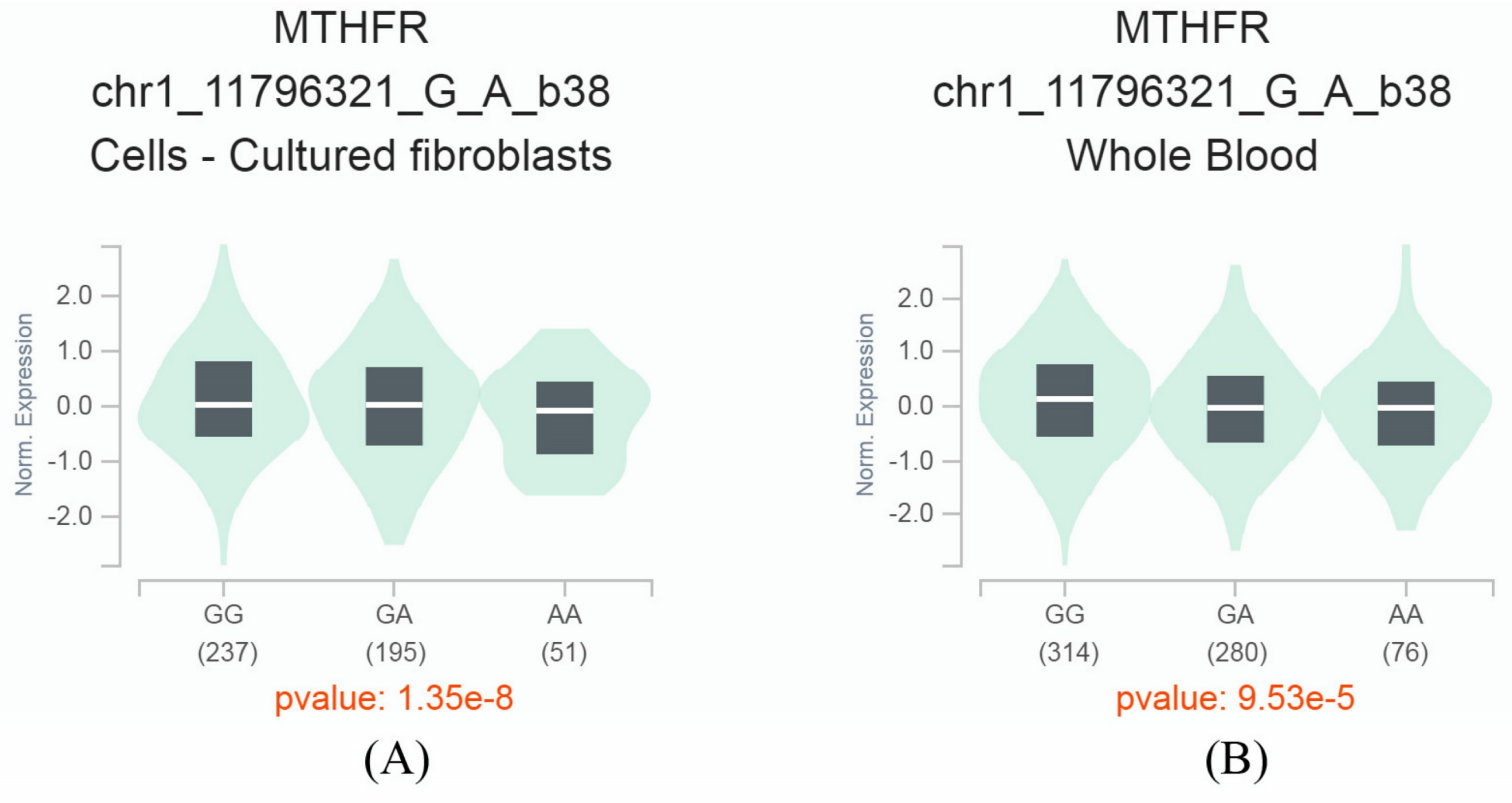
The *MTHFR* C677T variant is an expression quantitative trait locus (eQTL). This eQTL modulates the expression levels of the *MTHFR* gene. The diagram demonstrates the impact of the *MTHFR* C677T variant on the *MTHFR* gene expression in cultured fibroblasts (A) and whole blood (B), highlighting its significance in understanding genetic regulation and its potential implications in various biological processes.

By searching, we retrieved an eQTL result from the GTEx database, indicating a significant association between the *MTHFR* C677T polymorphism and the expression level of the *MTHFR* gene. The SNP is located at position 11796321 on chromosome 1 and has two allelic variants, C (G) and T (A). In cultured fibroblasts and human blood samples, we observed significant downregulation of gene *MTHFR* expression in carriers of the A allele compared with carriers of the G allele (*P*<0.001) (**Figure 4**). These findings suggest that the SNP may participate in regulating the *MTHFR* gene, and further functional studies could help elucidate this phenomenon.

## Discussion

This updated meta-analysis comprehensively assessed the association between *MTHFR* gene polymorphisms (C677T and A1298C) and susceptibility to NHL. Our overall analysis did not reveal a significant correlation between these SNPs and NHL risk. Given the significant evidence of an association of NHL risk with the two *MTHFR* SNPs in some studies 12, 21, 22, the contradictory findings may result from the heterogeneous nature of NHL, population ancestry, and source of controls. To control for potential confounding effects, we conducted stratified analyses to better understand the potential associations between *MTHFR* gene polymorphisms and NHL risk in specific subgroups. Stratified analysis revealed that the C677T polymorphism was significantly associated with increased NHL risk in Caucasians and the FL subtype but not in Asians or the DLBCL subtype. Moreover, FPRP analysis confirmed that these significant associations had low FPRP values, indicating a high probability that the associations are true positives and are robust against false-positives. These results are consistent with those of a previous meta-analysis by He *et al.* 15. Notably, they also reported a significant reversal association in Asians, with 3 studies included 15, whereas the current meta-analysis with 7 studies in Asians showed no evidence of this association. The paradoxical nature of these findings indicates that they may be the result of chance. The observed association between the C677T polymorphism and increased NHL risk in Caucasians underscores the potential ethnicity-specific effects of this genetic variant on NHL pathogenesis.

Moreover, the significant association in subgroups aligns with previous studies, which implicates the C677T polymorphism in altered folate metabolism, which may contribute to lymphomagenesis through mechanisms such as DNA methylation and nucleotide synthesis 10. DNA methylation and synthesis rely heavily on the accessibility of one-carbon, methyl-donating nutrients, and insufficiencies in nutrients such as folate or vitamins B6 and B12 might increase the risk of gene mutations and DNA double-strand breaks 11. A shortage of folate has been linked to several malignancies 47-49. Consistently, several studies have revealed that genetic polymorphisms in one-carbon-metabolizing pathway genes and folate-metabolizing genes, such as thymidylate synthase, serine hydroxymethyltransferase, methionine synthase, and MTHFR, can modify NHL predisposition 9, 12, 24, 25, 30, 31, 41.

The *MTHFR* C677T polymorphism is known to be linked to decreased *MTHFR* enzyme activity and lower plasma folate levels, leading to hypomethylation 10. It is reasonable that the *MTHFR* C677T polymorphism is associated with the risk of FL but not DLBCL. FL and DLBCL are the most common indolent and aggressive lymphomas, respectively; therefore, they are two distinct diseases with different environmental and genetic risk factors 2. For example, different progressively acquired DNA alterations (e.g., gene mutation, amplification or deletion and chromosomal translocation) may contribute to the development of different subtypes of NHL, such as the causal relationship between BCL2 translocation and FL or MYC translocation and Burkitt lymphoma 50. Studies conducted previously proposed a potential link between hypomethylation of the BCL-2 gene and the onset of this translocation 51. One possible mechanism may be that the *MTHFR* C677T polymorphism results in increased global DNA hypomethylation, which in turn induces chromosomal instability through the loss of epigenetic regulation and the activation of transposable elements. Such genomic instability may predispose B lymphocytes to BCL-2 gene translocation, thereby facilitating the development of FL.

Moreover, the *MTHFR* A1298C polymorphism did not significantly affect NHL risk in either the overall pooled analysis or the stratified analyses of our meta-analysis. He and colleagues demonstrated that the A1298C polymorphism significantly increased NHL susceptibility among Asians in a previous meta-analysis in which 3 studies were included 15. Intriguingly, the significance of the association disappeared in the currently updated meta-analysis, with 7 studies conducted in the Asian population. However, the potential role of the A1298C polymorphism in NHL susceptibility cannot be ruled out entirely. The lack of significance in our analysis may be attributed to the complex interplay of genetic and environmental factors influencing NHL development, highlighting the need for further investigation into the functional implications of the A1298C polymorphism in lymphomagenesis.

Overall, several unique aspects of our study may contribute to its originality and scientific value: 1) Inclusion of recent studies: The latest published meta-analysis on *MTHFR* SNPs and NHL susceptibility was performed in 2014. It has been ten years since then. Our meta-analysis integrated the latest available data, including several recent studies that have not been incorporated into previous analyses. This allows for an updated and comprehensive assessment of the SNPs in question, providing a more current understanding of their association with NHL. 2) Methodological Improvements: Our meta-analysis employed FPRP analysis to address possible false associations of SNPs with NHL susceptibility. Our FPRP results confirmed that the associations between the *MTHFR* gene C677T polymorphism and NHL risk were notable in some subgroups. These methodological enhancements increase the reliability of the conclusions drawn. 3) GTEx analysis: By exploring the GTEx database, we found that the *MTHFR* C677T polymorphism is an eQTL that is significantly correlated with alterations in *MTHFR* gene expression. This finding suggests that the SNP may participate in regulating the *MTHFR* gene. 4) Clinical Relevance: The findings from our updated meta-analysis have implications for personalized medicine, potentially guiding genetic screening and risk assessment strategies in clinical settings. Highlighting the translational aspect of our research underscores its practical relevance and novelty.

However, certain limitations should be acknowledged. The included studies varied in design, genotyping methods, sample size, and adjustment for confounding factors, which may have introduced heterogeneity and biased our results. Moreover, because only one study conducted with African participants was included, this meta-analysis was insufficient to estimate the risk effects of *MTHRF* SNPs among this population. Additionally, gene‒gene and gene‒environment interactions were not explored in this meta-analysis, warranting future research to elucidate the intricate mechanisms underlying NHL susceptibility associated with *MTHFR* gene polymorphisms.

In conclusion, our updated meta-analysis highlights the potential significance of the *MTHFR* gene C677T polymorphism in NHL risk, particularly among individuals of Caucasian ethnicity and in the FL subtype. These findings contribute to our understanding of the genetic basis of NHL and may help to foster risk stratification and personalized prevention strategies. Further large-scale studies and functional analyses are needed to validate our findings and elucidate the underlying biological mechanisms involved.

## Figures and Tables

**Table 1:** Characteristics of studies included in the final meta-analysis for the association between *MTHFR* C677T and A1298C polymorphisms and NHL risk

Surname	Year	Country	Ethnicity	Source	Genotype method	Case				Control				MAF	HWE
						WW	WM	MM	All	WW	WM	MM	All		
**C677T polymorphism**															
Gonzalez Ordonez	2000	Spain	Caucasian	HB	PCR-RFLP	21	21	5	47	92	88	20	200	0.32	0.876
Lincz	2003	Australia	Caucasian	HB	PCR-RFLP	73	58	17	148	145	133	21	299	0.29	0.198
Toffoli	2003	Italy	Caucasian	PB	PCR-RFLP	44	49	18	111	147	233	85	465	0.43	0.662
Gemmati	2004	Italy	Caucasian	PB	PCR-RFLP	60	101	39	200	78	128	51	257	0.45	0.908
Linnebank	2004	German	Caucasian	PB	PCR-RFLP	13	12	6	31	66	52	24	142	0.35	0.019
Matsuo	2004	Japan	Asian	HB	PCR-RFLP	165	122	63	350	182	230	88	500	0.41	0.301
Rudd	2004	UK	Caucasian	HB	Taqman	361	381	90	832	383	397	106	886	0.34	0.841
Skibola	2004	USA	Caucasian	PB	Taqman	122	160	52	334	288	350	84	722	0.36	0.149
Lightfoot	2005	UK	Caucasian	PB	Taqman	247	270	72	589	356	316	83	755	0.32	0.309
Stanulla	2005	German	Caucasian	PB	PCR-RFLP	207	216	64	487	184	152	43	379	0.31	0.179
Chen	2006	China	Asian	HB	Taqman	11	13	4	28	72	66	19	157	0.33	0.522
Deligezer	2006	Turkey	Caucasian	HB	Taqman	31	30	5	66	66	72	16	154	0.34	0.574
Niclot	2006	France	Caucasian	PB	DHPLC	66	86	20	172	92	88	24	204	0.33	0.674
Timuragaoglu	2006	Turkey	Caucasian	PB	Realtime PCR	31	22	5	58	36	36	10	82	0.34	0.829
Lee	2007	Australia	Caucasian	PB	Taqman	253	227	74	554	256	190	57	503	0.30	0.019
Lim	2007	USA	Mixed	PB	Taqman	499	477	127	1103	443	396	86	925	0.31	0.853
Siraj	2007	Saudi Arabia	Caucasian	PB	PCR-RFLP	109	45	6	160	372	126	13	511	0.15	0.553
Gra	2008	Russia	Caucasian	HB	Hybridization	39	28	9	76	85	79	13	177	0.30	0.354
Kim	2008	Korea	Asian	PB	PCR-RFLP	223	286	75	584	540	863	297	1700	0.43	0.133
Berglund	2009	Sweden	Caucasian	PB	Illumina	154	85	24	263	241	157	32	430	0.26	0.363
Ismail	2009	Jordan	Caucasian	PB	PCR-RFLP	34	19	2	55	94	66	10	170	0.25	0.722
Wang	2009	Jamaica	Mixed	PB	Taqman	329	58	5	392	204	57	5	266	0.13	0.664
Kurzwelly	2010	German	Caucasian	PB	PCR-RFLP	78	81	26	185	96	96	20	212	0.32	0.568
Weiner	2011	Russia	Caucasian	PB	Taqman	72	60	11	143	242	198	46	486	0.30	0.553
Li	2013	USA	Mixed	PB	Taqman	202	206	72	480	236	246	82	564	0.36	0.173
Ayad	2014	Egypt	African	PB	PCR-RFLP	19	24	6	49	53	23	6	82	0.21	0.136
Suthandiram	2014	Malaysia-Malay	Asian	PB	MassARRAY	144	49	6	199	236	66	5	307	0.12	0.876
Suthandiram	2014	Malaysia-Chinese	Asian	PB	MassARRAY	67	48	6	121	155	98	12	265	0.23	0.479
Suthandiram	2014	Malaysia-Indian	Asian	PB	MassARRAY	45	7	0	52	128	20	2	150	0.08	0.249
Bradshaw	2015	Australia	Caucasian	HB	PCR-RFLP	97	85	25	207	88	94	19	201	0.33	0.393
Fragkioudaki	2017	Greece	Caucasian	HB	PCR-RFLP	4	10	5	19	235	291	74	600	0.37	0.268
Mashhadi	2018	Iran	Asian	PB	TARMS-PCR	82	42	3	127	150	53	2	205	0.14	0.252
**A1298C polymorphism**															
Lincz	2003	Australia	Caucasian	HB	PCR-RFLP	64	68	13	145	124	139	31	294	0.34	0.385
Toffoli	2003	Italy	Caucasian	PB	PCR-RFLP	54	44	13	111	200	222	43	465	0.33	0.094
Gemmati	2004	Italy	Caucasian	PB	PCR-RFLP	96	90	14	200	126	110	21	257	0.30	0.659
Linnebank	2004	German	Caucasian	PB	PCR-RFLP	16	12	3	31	69	54	19	142	0.32	0.116
Matsuo	2004	Japan	Asian	HB	PCR-RFLP	209	122	19	350	327	150	23	500	0.20	0.282
Rudd	2004	UK	Caucasian	HB	Taqman	397	363	72	832	412	389	85	886	0.32	0.622
Skibola	2004	USA	Caucasian	PB	Taqman	178	128	27	333	341	310	71	722	0.31	0.964
Lightfoot	2005	UK	Caucasian	PB	Taqman	288	250	51	589	347	331	77	755	0.32	0.882
Niclot	2006	France	Caucasian	PB	DHPLC	79	76	17	172	102	81	15	198	0.28	0.844
Lim	2007	USA	Mixed	PB	Taqman	540	480	104	1124	461	393	81	935	0.30	0.831
Siraj	2007	Saudi Arabia	Caucasian	PB	PCR-RFLP	38	40	35	113	239	220	52	511	0.32	0.896
Gra	2008	Russia	Caucasian	HB	Hybridization	36	30	10	76	81	82	14	177	0.31	0.278
Kim	2008	Korea	Asian	PB	Taqman	372	182	29	583	1147	500	53	1700	0.18	0.868
Berglund	2009	Sweden	Caucasian	PB	Illumina	116	121	25	262	214	196	39	449	0.31	0.533
Ismail	2009	Jordan	Caucasian	PB	PCR-RFLP	20	23	12	55	76	81	13	170	0.31	0.172
Wang	2009	Jamaica	Mixed	PB	Taqman	277	98	15	390	201	65	9	275	0.15	0.198
Kurzwelly	2010	German	Caucasian	PB	PCR-RFLP	72	96	17	185	106	89	17	212	0.29	0.779
Weiner	2011	Russia	Caucasian	PB	Taqman	59	52	22	133	232	215	56	503	0.33	0.562
Li	2013	USA	Mixed	PB	Taqman	246	203	40	489	265	250	59	574	0.32	0.997
Jiang	2014	China	Asian	HB	Taqman	17	9	2	28	109	46	2	157	0.16	0.238
Suthandiram	2014	Malaysia-Malay	Asian	PB	MassARRAY	104	82	13	199	137	147	23	307	0.31	0.052
Suthandiram	2014	Malaysia- Chinese	Asian	PB	MassARRAY	74	40	7	121	160	85	20	265	0.24	0.073
Suthandiram	2014	Malaysia-Indian	Asian	PB	MassARRAY	11	27	14	52	57	75	18	150	0.37	0.375
Bradshaw	2015	Australia	Caucasian	HB	HRM	94	92	25	211	93	85	24	202	0.33	0.502
Fragkioudaki	2017	Greece	Caucasian	HB	PCR-RFLP	15	4	0	19	273	266	61	600	0.32	0.747
Mashhadi	2018	Iran	Asian	PB	TARMS-PCR	69	40	18	127	110	69	26	205	0.30	0.006

NHL, non-Hodgkin lymphoma; MAF, Minor allele frequency; HWE, Hardy-Weinberg equilibrium; W, wild type; M, mutant type; HB, Hospital based; PB, Population based; PCR-RFLP, Polymorphism chain reaction-restriction fragment length polymorphism; TARMS-PCR, Tetra Amplification Refractory Mutation System polymerase chain reaction; HRM, high resolution melt; DHPLC, Denaturing high performance liquid chromatography.

**Table 2:** Meta-analysis for the association between *MTHFR* C677T and A1298C polymorphisms and non-Hodgkin lymphoma risk

Variables	No. of	Homozygous		Heterozygous		Recessive		Dominant		Allele Comparing	
	studies	OR (95% CI)	*P* ^het^	OR (95% CI)	*P* ^het^	OR (95% CI)	*P* ^het^	OR (95% CI)	*P* ^het^	OR (95% CI)	*P* ^het^
**C677T** (rs1801133)		TT vs. CC		CT vs. CC		TT vs. (CT + CC)		(CT + TT) vs. CC		T vs. C	
All	32	1.10 (0.96-1.24)	0.088	1.00 (0.92-1.10)	0.010	1.06 (0.97-1.17)	0.538	1.02 (0.94-1.12)	0.001	1.04 (0.97-1.11)	0.001
Ethnicity											
Caucasian	21	**1.16 (1.02-1.32)**	0.626	1.05 (0.97-1.14)	0.591	1.12 (1.00-1.26)	0.790	1.07 (0.99-1.16)	0.479	**1.07 (1.01-1.13)**	0.460
Asian	7	0.83 (0.60-1.15)	0.242	0.97 (0.74-1.26)	0.014	0.85 (0.69-1.04)	0.337	0.98 (0.75-1.27)	0.008	1.00 (0.82-1.21)	0.020
Mixed	3	1.16 (0.92-1.46)	0.364	0.92 (0.71-1.19)	0.067	1.15 (0.93-1.43)	0.473	0.93 (0.71-1.22)	0.034	0.96 (0.76-1.20)	0.023
African	1	2.79 (0.80-9.71)	/	**2.91 (1.34-6.32)**	/	1.77 (0.54-5.82)	/	**2.89 (1.39-6.00)**	/	**2.14 (1.23-3.73)**	/
Source of control											
HB	9	1.04 (0.82-1.32)	0.299	0.87 (0.72-1.05)	0.144	1.07 (0.89-1.29)	0.429	0.91 (0.77-1.07)	0.204	0.97 (0.87-1.08)	0.312
PB	23	1.11 (0.95-1.29)	0.078	1.05 (0.95-1.16)	0.040	1.06 (0.96-1.18)	0.487	1.06 (0.96-1.18)	0.003	1.06 (0.97-1.11)	0.001
Subtype											
DLBCL	15	0.98 (0.83-1.15)	0.077	0.99 (0.83-1.18)	0.002	1.03 (0.88-1.20)	0.269	1.01 (0.86-1.19)	0.001	1.03 (0.92-1.16)	0.011
FL	12	**1.25 (1.02-1.53)**	0.655	0.91 (0.75-1.10)	0.081	**1.28 (1.06-1.56)**	0.721	0.97 (0.82-1.15)	0.133	1.06 (0.95-1.17)	0.359
**A1298C** (rs1801131)		CC vs. AA		AC vs. AA		CC vs. (AC + AA)		(AC + CC) vs. AA		C vs. A	
All	26	1.20 (0.99-1.47)	<0.001	1.00 (0.94-1.07)	0.279	1.20 (1.00-1.44)	<0.001	1.04 (0.95-1.13)	0.011	1.07 (0.98-1.17)	<0.001
Ethnicity											
Caucasian	16	1.20 (0.92-1.59)	<0.001	0.97 (0.89-1.07)	0.281	1.21 (0.93-1.58)	<0.001	1.02 (0.90-1.16)	0.022	1.06 (0.94-1.21)	<0.001
Asian	7	1.39 (0.91-2.12)	0.047	1.08 (0.95-1.24)	0.247	1.34 (0.95-1.89)	0.153	1.11 (0.91-1.36)	0.086	1.13 (0.95-1.36)	0.032
Mixed	3	0.96 (0.72-1.29)	0.293	1.00 (0.87-1.14)	0.471	0.98 (0.77-1.24)	0.426	0.99 (0.85-1.15)	0.291	0.99 (0.86-1.14)	0.199
Source of control											
HB	7	1.04 (0.76-1.42)	0.264	1.01 (0.88-1.15)	0.192	1.02 (0.78-1.34)	0.335	1.00 (0.81-1.24)	0.083	1.02 (0.86-1.21)	0.056
PB	19	1.25 (0.98-1.58)	<0.001	1.00 (0.93-1.08)	0.335	1.24 (0.99-1.55)	<0.001	1.04 (0.94-1.16)	0.019	1.09 (0.98-1.21)	<0.001
Subtype											
DLBCL	12	1.21 (0.87-1.69)	0.003	1.01 (0.90-1.13)	0.738	1.24 (0.89-1.72)	0.001	1.04 (0.92-1.17)	0.237	1.07 (0.98-1.16)	0.002
FL	11	1.23 (0.91-1.67)	0.198	1.04 (0.90-1.20)	0.128	1.19 (0.93-1.53)	0.365	1.08 (0.88-1.32)	0.056	1.07 (0.96-1.19)	0.041

HB, Hospital based; PB, Population based; DLBCL, diffuse large B-cell lymphoma; FL, follicular lymphoma.

**Table 3:** False-positive report probability analysis for the associations between *MTHFR* gene C677T polymorphism and non-Hodgkin lymphoma risk

Genotype	OR (95% CI)	*P* ^a^	Statistical Power ^b^	Prior probability				
				0.25	0.1	0.01	0.001	0.0001
TT vs. CC								
Caucasian	1.16 (1.02-1.32)	0.022	1.000	**0.062**	**0.165**	0.685	0.956	0.995
FL	1.25 (1.02-1.53)	0.033	0.980	**0.092**	0.233	0.769	0.971	0.997
CT vs.CC								
African	2.91 (1.34-6.32)	0.007	0.053	0.284	0.544	0.929	0.993	0.999
TT vs. CT/CC								
FL	1.28 (1.06-1.56)	0.011	0.953	**0.033**	**0.094**	0.533	0.920	0.991
CT/TT vs. CC								
African	2.89 (1.39-6.00)	0.005	0.045	0.250	0.500	0.917	0.991	0.999
T vs. C								
Caucasian	1.07 (1.01-1.13)	0.023	1.000	**0.065**	**0.171**	0.695	0.958	0.996
African	2.14 (1.23-3.73)	0.007	0.128	**0.141**	0.329	0.844	0.982	0.998

OR, odds ratio; CI, confidence interval; FL, follicular lymphoma. ^a^ Chi-square test was used to calculate the genotype frequency distributions. ^b^ Statistical power was calculated using the number of observations in the subgroup and the OR and *P* values in this table.
